# Regulatory T cell therapy for Sjögren's disease: From pathogenesis to targeted treatment

**DOI:** 10.1016/j.jtauto.2025.100311

**Published:** 2025-08-26

**Authors:** Zhi Feng Sherman Lim, Alberta Y. Hoi, Fabien B. Vincent, Joshua D. Ooi, Eric F. Morand, Maureen Rischmueller, Yi Tian Ting

**Affiliations:** a Centre for Inflammatory Diseases, Department of Medicine, Monash University, Clayton, Victoria, Australia; b Rheumatology Department, Monash Health, Clayton, Victoria, Australia; c University of Adelaide, Faculty of Health and Medical Sciences, Adelaide, South Australia, Australia; d Rheumatology Department, The Queen Elizabeth Hospital, Woodville South, South Australia, Australia

**Keywords:** Regulatory T cells (Treg), Autoimmunity, Cell therapy, HLA, SSA/Ro, Sjögren's disease (SjD)

## Abstract

Sjögren's disease (SjD) is a chronic systemic autoimmune disorder characterised by lymphocytic infiltration of the salivary and lacrimal glands, leading to the hallmark symptoms of dry eyes and dry mouth. Beyond glandular dysfunction, many patients experience systemic complications—including B cell hyperactivity, organ-specific inflammation, and a markedly increased risk of non-Hodgkin lymphoma—that are frequently under-recognised and poorly managed. Current treatments remain largely empirical and symptomatic, with limited efficacy in modifying disease progression or restoring immune tolerance.

Recent advances have illuminated profound dysregulation in both innate and adaptive immunity, revealing novel therapeutic targets now under investigation in clinical trials, including type I interferon signalling, B cell activation, and co-stimulatory pathways. Central to this dysregulation is T cell–driven pathology: CD8^+^ T cell cytotoxicity, defective regulatory T cell (Treg) function, and HLA class II–mediated presentation of self-antigens to autoreactive CD4^+^ T cells are key mechanisms in disease initiation and persistence.

A growing body of evidence implicates Ro autoantigens—Ro60 and Ro52—as central targets in SjD pathogenesis. Anti-Ro antibodies are present in approximately 70 % of patients and serve as both diagnostic markers and indicators of systemic involvement. Ro antigens and their corresponding antibodies are consistently detected in inflamed salivary tissues, underscoring their potential as compelling targets for antigen-specific therapy.

This review examines the immunopathogenic role of Ro-specific T cell responses in SjD and outlines how engineered Treg-based therapies may enable precise immune modulation, restore tolerance, and provide durable disease control for patients with this complex autoimmune condition.

## Highlights

• Rising prevalence of Sjögren's contrasts with limited targeted interventions.
• T cell subset dysfunction, particularly in regulatory T cells, drives pathology.
• HLA-DR3 and Ro autoantigens inform genetic risk and antigen-specific therapy.
• Current therapies target B cells and epithelial cells with varying efficacy.
• Advances in antigen-specific and engineered Treg therapies offer new avenues.

## 1 Overview of Sjögren's disease

Sjögren's disease (SjD) is a relatively common autoimmune condition, affecting about 0.2 % of adults worldwide [1]. It is the second most prevalent systemic autoimmune condition in the United States and the United Kingdom [2,3]. It frequently coexists with other autoimmune diseases, with a 20 % increase in diagnoses over the past two decades due to improved recognition [2,3]. SjD disproportionately affects women, with a global female-to-male ratio of 13:1, particularly in individuals aged 50 to 60 [[4], [5], [6], [7], [8]]. This sex disparity also varies greatly according to ethnicity, being more pronounced in people of Asian ethnicity (27:1), while African Americans have the lowest known ratio (7:1) [3]. Moreover, female patients with SjD often experience more severe systemic and immunological symptoms with distinct phenotypes compared to male individuals [4,5].

SjD primarily targets the exocrine glands, causing chronic lymphocytic infiltration and dysfunction of the salivary and lacrimal glands in particular, which leads to the hallmark *sicca* complex [4,5,[8], [9], [10]]. Approximately 95 % of patients report xerostomia (dry mouth), dry eyes, and dryness of other mucosal surfaces (e.g., vaginal, trachea) [5,7,8,11]. Reduced salivary production can cause dysgeusia (altered taste), oral pain, burning sensations, and difficulties with swallowing and speaking, especially after prolonged periods without fluid intake [5]. Oral examination often reveals erythematous mucosa, a depapillated tongue, dental caries, gum recession, periodontal disease, bacterial and fungal infections [12,13]. Glandular swelling, affecting the parotid, submandibular, sublingual or lacrimal glands, occurs in about 30 % of patients [5,14].

Beyond dryness, patients frequently experience constutional symptoms such as fever, night sweats, or weight loss, and a subset of patients are beset by severe fatigue and/or joint and muscular pain. All these symptoms can significantly impact patients’ quality of life, including social function, work capacity, and are associated with economic burden as a consequence of dental and over-the-counter medication costs. The exact aetiology and mechanisms of fatigue and “non-inflammatory” pain in SjD have not been elucidated, and unlike systemic disease in SjD are not typically associated with peripheral B cell or type 1 interferon signatures. Whether their biological underpinnings are shared across other connective tissue diseases or specific to SjD remains unknown, and other factors such as sleep disturbance, autonomic nervous system dysfunction and mood disorders may contribute [5,15].

Less well-recognised are the extra-glandular manifestations, where people with SjD may experience localised or systemic inflammation. The most common target end organs are musculoskeletal and cutaneous, but overall, the spectrum of clinical manifestations is broad. Various attempts have been made to simplify this, for example, Mihai et al. have proposed seven “systems”, such as musculoskeletal, dermatologic, neurologic, renal, haematologic, pulmonary and gastrointestinal This has been further elaborated by EULAR Sjögren's Syndrome Disease Activity Index (ESSDAI) [16], which comprising 12 domains including glandular enlargement [17](Table 1).

**Table 1:** Organ involvement in SjD organised by ESSDAI domains.

*Systems*	Clinical manifestation
**Constitutional**	Fever, night sweats and/or weight loss
**Lymphadenopathy**	Lymphadenopathy and/or splenomegaly, B cell lymphoma
**Glandular**	Parotid, submandibular or lacrimal gland enlargement
**Articular**	Inflammatory arthralgia or synovitis
**Dermatologic**	Erythema multiforme, subacute cutaneous lupus, urticarial vasculitis, leukocytoclastic vasculitis
**Pulmonary**	Bronchial involvement, interstitial lung disease, or small airway disease
**Renal**	Renal tubular acidosis, glomerulonephritis or interstitial nephritis, cryoglobulinemic vasculitis
**Muscular**	Myositis with or without weakness
**Peripheral nervous system**	Axonal polyneuropathy (sensory only or sensorimotor), ganglionopathy, demyelinating polyneuropathy, vasculitic neuropathy, small fibre neuropathy, or trigeminal neuralgia.
**Central nervous system**	Optic neuritis, multiple sclerosis like syndrome, cognitive impairment, cerebral vasculitis with stroke-like presentation, seizure, transverse myelitis, meningitis.
**Haematologic**	Neutropenia, lymphopenia, anaemia, thrombocytopenia
**Biological**	Hypergammaglobulinemia, hypocomplementemia, cryoglobulinemia (and recent onset of hypogammaglobulinemia)

Systemic involvement, as defined by activity in any ESSDAI domain, was present at the time of diagnosis in 81.8 % of patients with SjD in a large international cohort (N > 10,000) (Brito-Zeron 2020). The mean total ESSDAI score at diagnosis of the entire cohort was 6.1 (moderate) (S.D. 7.5); The domains with the highest frequency of active patients included the biological (51 %), articular (37.7 %), haematological (22.4 %), glandular (21.4 %) and pulmonary (10.4 %) domains. Males with SjD had higher mean ESSDAI (8.1 vs 6.0, P < 0.001) scores, and a higher frequency of high disease activity (22.5 % vs 11.7 %, P < 0.001) compared with females. The organ-specific ESSDAI domains that showed significantly increased activity in males compared with females included the lymphadenopathy (P < 0.001), glandular (P < 0.001), pulmonary (P < 0.001), peripheral nervous system (PNS) (P < 0.001) and CNS (P < 0.001) domains.

It remains uncertain whether patients with SjD have a lower life expectancy, however mortality rate in SjD is partly driven by the heightened risk of malignancies, including the well-recognised risk of non-Hodgkin lymphoma. Several clinical features have been identified as being associated with increased lymphoma risk, in addition to the high ESSDAI score. Independent predictors for lymphoma development include salivary gland enlargement, lymphadenopathy, Raynaud phenomenon, anti-Ro/SSA or/and anti-La/SSB autoantibodies, rheumatoid factor (RF) positivity, monoclonal gammopathy, and C4 hypocomplementemia [18,19]. Furthermore, studies also shown that patients with SjD have an increased risk of overall malignancies, and not solely attributable to lymphoma risk [20]. The key mortality risk factors at the time of SjD diagnosis were positive cryoglobulins and a high systemic activity scored using the ESSDAI, conferring a 2-times increased risk of all-cause and SjD-related death (12). High systemic activity at diagnosis was also related to poor survival due to infections and cardiovascular disease.

In addition to the malignancy risk, 14 % of deaths were directly attributed to systemic SjD, often involving severe complications such as renal failure, systemic vasculitis, and pulmonary fibrosis [11]. Similar to other complex multiorgan autoimmune diseases, 60 % of deaths were due to infections and cardiovascular diseases unrelated to SjD, highlighting the multifaceted impact of the disease on patient outcomes [11].

Traditionally, SjD has been classified as either primary or more commonly secondary, when it coexists with other autoimmune disorders, most notably SLE, RA or systemic sclerosis (SSc) [4,6,10,21]. The similarities between primary and secondary SjD have been highlighted in multiple studies, including clinical, immunological and histological findings. Currently, the distinction between primary and secondary SjD does not affect therapeutic strategies [21]. Studies are underway to investigate the significance of various immunological pathways and cellular mechanisms to better understand the distinct processes driving SjD in different clinical contexts [22].

## 2 Pathogenesis of SjD

T cells play a central role in the pathogenesis of SjD which is characterized by early lymphocytic infiltration in the exocrine glands by activated CD4^+^ T cells [8]. Their involvement, together with other T cell subsets, contributed to glandular epithelial cell damage and formation of ectopic lymphoid structures [23]. This T cell-driven response is genetically determined as it requires presentation of self-antigens via their major histocompatibility complex (MHC) class II molecules to T cell receptors [24]. The autoimmune response may be influenced by the specific variants of MHC Class II molecules and/or exact autoepitopes, and may be further amplified by environmental factors, such as viral infections (e.g., cytomegalovirus (CMV), Epstein-Barr virus (EBV)) [25,26]. We will discuss how these interactions with T cells and other immune pathways promote the perpetuation of loss of central and peripheral tolerance and the immune polarisations observed in SjD and production of pathogenic autoantibodies by B cells.

Genetic factors play a substantial role in the development of SjD, with several studies linking the inheritance of specific MHC genes to the condition (Table 2) [8]. Notably, 90 % of patients with SjD carry the HLA-DRB1∗03:01 gene, encoding for an MHC Class II molecule commonly found in European and Chinese populations [5,6,24,[27], [28], [29], [30], [31], [32]]. A study by Kang et al. found that both USA (California) and Chinese patients with SjD had a significantly higher frequency of HLA-DRB1∗03:01 compared to healthy individuals (*p* < 0.001, p < 0.05) [29]. Similarly, a study of 206 Chinese nationals showed that HLA-DRB1∗03:01 was more significantly prevalent in patients with SjD than in healthy controls [30]. Other studies carried out in Colombia, France, Hungary and Australia have also reported a link between HLA-DRB1∗03:01 and SjD [[31], [32], [33], [34]].

**Table 2:** Ethnicity studies in SjD.

Study	Population	SjD (n)	HC (n)	Statistic	Type of Study	Reference
**Kang Ho Li et al (1993)**	American Caucasian (California)	75	135	p < 0.001	Molecular	[29]
**Kang Ho Li et al (1993)**	China	45	42	p < 0.05	Molecular	[29]
**Wang J et al (1997)**	China	70	136	p < 0.005, RR:4.56	Serological	[30]
**Anaya JM et al (2002)**	Columbian	73	76	p = 0.001, OR: 15.5	Molecular	[32]
**Gottenberg JE et al (2003)**	French	149	222	RR: 3.8	Molecular	[35]
**Kóvacs A et al (2006)**	Hungarian	48	50	p < 0.02	Molecular	[31]
**Rischmuller M et al (1998)**	Australian	80	97	p < 0.0001	Molecular	[34]
**Nakken. B et al (2008)**	Norwegian	15	45	p = 0.0078	Molecular	[36]

**Fig. 1:**
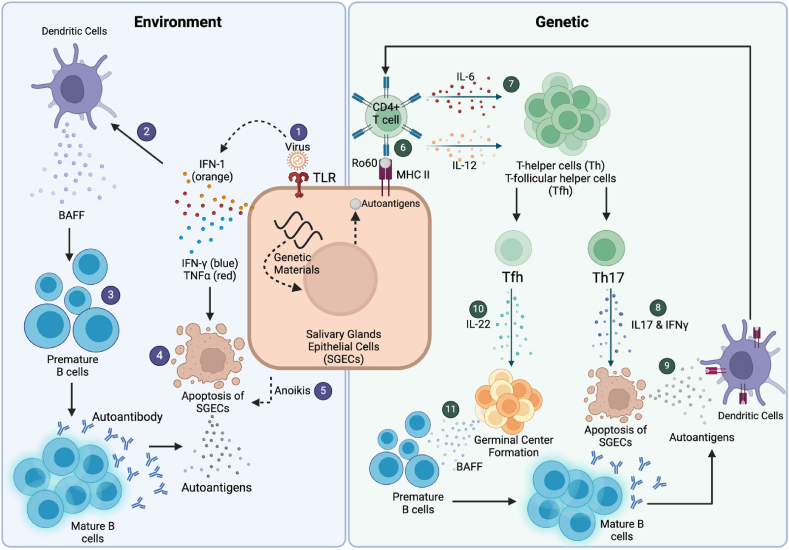
Schematic representation showing the potential role of EBV and other viral infection (Environment) and MHC Class II involvement (Genetic) in SjD pathogenesis. Environment; (1) EBV is recognised by TLR3 causing production of Type 1 interferons (IFN) and other proinflammatory cytokines such TNFα and IFNγ. (2) Type 1 IFN stimulate myeloid dendritic cells to produce BAFF causing (3) B cells to mature and produce autoantibodies targeting autoantigens. (4) Proinflammatory cytokines will cascade maturation of B cells and apoptosis of SGECs releasing autoantigens. (5) Through TLR3 activation, anoikis of SGECs cause the release of autoantigens for autoantibody recognition. Anoikis is a type of programmed cell death where cell detached from its extracellular matrix disrupting integrin ligation [44]. Genetic; (6) Extracellular or intracellular Ro60 will be processed and presented on the MHC Class II molecule of SGECs, acting as a non-professional antigen presenting cell, to CD4^+^ T cells causing recognition of the complex via its TCR that led to the production of cytokines IL-6 and IL-12. (7) These cytokines initiate differentiation of T cells to Ro60-specific T helper cells (Th -1 and -17) and T follicular helper (Tfh). (8) Th-17 produce IL-17 and IFNγ causing apoptosis of SGECs. (9) Ro60 would be released from SGECs and led to the uptake by mDCs and presented via their MHC Class II molecules. (10) Tfh produces IL-22 causing activation of germinal center to secrete BAFF. (11) BAFF promote maturation of B cells leading to the production of autoantibody targeting Ro60. (Figure created using Biorender).

The HLA-DRB01∗03:01 allele plays a critical role in antigen presentation, and variations in the HLA gene sequence affects the repertoire of peptides that can be bound and presented to the immune system. In SjD, the HLA-DRB1∗03:01 variant facilitates the presentation of self-antigens derived from salivary gland epithelial cells (SGECs) to autoreactive T cells, contributing to the breakdown of immune tolerance and the initiation of autoimmune responses characteristic of the disease. (Fig. 1). Apoptosis of SGECs is reported to be a major source of self-antigens [[37], [38], [39]]. In addition to the SGEC, another major source of Ro antigens is from the cytoplasm of epidermal cells especially following UV irradiation [40]. When these self-antigens are presented on MHC Class II by plasmacytic and myeloid DCs (pDC and mDC), CD4^+^ T cells will recognise the autoantigen through its T cell receptor (TCR) [37,38]. Other autoantigens have been identified that may lead to findings of novel autoantibodies in this condition [[41], [42], [43]]. Activation of CD4^+^ T cells then leads to proliferation and differentiation into a number of well-defined T cell subsets such as T helper 1 (Th1), T helper 17 (Th17), T follicular helper (Tfh) and T regulatory cells [4,37] (Fig. 1).

In addition to DCs, SGECs could act as non-professional antigen presenting cells (APC) as they express high level of HLA-DR and costimulatory molecules such CD80 and CD86 [44]. SGECs may present self-antigens to CD4^+^ T cells, triggering the production of cytokines, including IL-6 and IL-12 [44,45]. These cytokines promote the differentiation of CD4^+^ T cells into Th1, Th17, and Tfh cells, which amplifies the immune response [4,8,21].

There is significant interaction between Th17 and Tfh cells which are distinct subsets of CD4 T cells that have shared cytokine networks [4]. Th17 cells produce IL-21 and IL-22, cytokines that contribute to inflammation and tissue damage [8]. IL-21 is also a hallmark cytokine of Tfh cells promoting their differentiation and function, particularly in germinal centre formation [8]. Both Th17 and Tfh cells share similar differentiation pathways influenced by IL-6 and IL-21, and transcription factors such as STAT3, with potential plasticity between these subsets [8,21]. Both subsets provide help to B cells within the germinal centres, aiding in B cell maturation, class switching and autoantibody production, under the influence of cytokines such as B cell-activating factor (BAFF) [4,21,46].

Tissue-resident CD8^+^ T cells are cytotoxic and can directly damage SGECs, further leading to the release of vesicles containing self-antigens, which can be taken up by DCs [4,8,21,46]. The cytotoxic T lymphocytes (CTLs) secrete cytokines such as IFNγ, granzyme and perforin and TNFα [8]. Interestingly, these CD8^+^ T cells can produce IL-17 and in an experimental model may contribute to CNS autoimmunity [47].

## 3 Key autoantibodies and autoantigens involved in SjD

Autoantibodies are a serological hallmark of Sjögren's disease (SjD), with variability in antibody profiles correlating with distinct clinical manifestations such as vasculitis and haematologic abnormalities (Table 3). Among these, anti-Ro and anti-La antibodies are highly prevalent and strongly associated with more severe systemic involvement, increased disease activity, and a higher risk of complications [48].

**Table 3:** Key autoantigens and clinical associations of their cognate autoAb in SjD.

Antigens	Cognate autoAb prevalence	Clinical Manifestation
**Ro52**	71 % [13], 73 % [14]	Extra-glandular [49], cutaneous lupus [50]
**Ro60**		
**La**	23 %–53 % [49]	Extra-glandular [49]
**M3R**	60 % [49]	Sicca symptoms [49]
**Rheumatoid Factor (RF)**	80 % [51]	Extra-glandular and lymphoma [51]
α**fodrin**	38 %–42 % [50]	Neurological [52], Hypergammaglobulinaemia [50]

Abbreviations: Antibody, Ab; M3 muscarinic receptor, M3R.

Anti-Ro antibodies, including both anti-Ro60 and anti-Ro52, are detected in approximately 70 % of SjD patients [13,14] and are widely used in clinical diagnostics [49,53,54]. Other autoantigens, such as La, M3R, and α fodrin, are also implicated in SjD, though their diagnostic utility is less pronounced. La, an RNA-binding phosphoprotein involved in RNA polymerase III transcript maturation, is associated with a prevalence of 23–53 % in SjD [41,49,55]. Anti-M3R, an autoantibody targeting a G-protein coupled receptor involved in glandular fluid secretion, has a prevalence of up to 60 %in SjD patients, though its diagnostic and pathogenic significance remains unclear due to inconsistent assay reproducibility [42,49,56]. Similarly, α fodrin, a cytoskeletal protein, shows a prevalence of 38–42 % but has limited sensitivity and specificity for SjD diagnostic [43,50,57,58]. Rheumatoid factor IgM is one of the important autoantibodies found in 80 % of SjD patients and it can also be detected in other autoimmune disease like Rheumatoid Arthritis (RA) and cryoglobulinemic vasculitis [51].

Recent research highlights anti-Ro52 and anti-Ro60 autoantibodies as valuable diagnostic and prognostic markers in SjD [59,60]. Ro60, a conserved RNA-binding protein, facilitates the degradation of misfolded RNA [41,61], while Ro52, a ubiquitin E3 ligase, regulates transcription and Type I interferon (IFN-1) production [10,60,62]. Emerging evidence suggests that isolated anti-Ro52 antibodies are linked to more severe phenotypes, including systemic involvement [59,63,64]. Differentiating between anti-Ro52 and anti-Ro60 detection enhances diagnostic precision, making it a potential marker for patient stratification in clinical trials [59,65]. Notably, anti-Ro antibodies can be detected in saliva but not in serum from patients classified as “seronegative”, accounting for up to 25 % of such cases [60,66,67].

Salivary diagnostics, currently used in research settings, may further enhance the utility of anti-Ro antibodies for early diagnosis and disease monitoring [60,66]. Additionally, animal model studies suggest that Ro peptides play a key role in disease initiation [68,69], creating opportunities for antigen-specific therapies. However, while targeting Ro and La autoantibodies or their associated pathways represents a promising therapeutic strategy, their exact contribution to disease pathology remains incompletely understood. Developing effective treatments requires a precise understanding of the specific regions targeted by these autoantibodies in each condition, a task further complicated by inter-patient variability.

Recent research has focused on identifying key autoantigens, including Ro60, Ro52, and La, where their cognate antibodies were found in the saliva and salivary glands of SjD patients [70,71]. T-cell epitope mapping efforts have pinpointed specific regions involved in autoimmune responses, such as the RNA-binding domain of Ro60 (amino acids 181–320) [59,64] and the coiled-coil/B-box domains of Ro52 (amino acids 1–240) [65,72](Table 4). In vivo studies using BALB/c mice demonstrate that specific Ro60 peptides can trigger autoimmune-like responses in salivary glands, underscoring their role in immune cell activation [68]. Furthermore, in another murine model, researchers were able to create a temporary immune reset by depleting CD4^+^ T cells, and then administer Ro peptides to promote the generation of Ro-specific Treg *in vivo* [73]. Adoptive transfer of these T regs into new mice could successfully increase salivary flow and suppress Th1 and B cell immune responses in the salivary glands [72]. These findings suggest that Ro antigens could serve as both biomarkers and therapeutic targets.

**Table 4:** Autoepitope mapping of autoantigens involved in SjD.

Antigen	Amino Acid Region	Methods	Reference
**Ro52**	Multiple epitopes: including those in regions for zinc finger domains	ELISA, immunoprecipitation	Lee et al., 2021 [64]
	175-197, 340-360	Multiplex bead assays, recombinant proteins	Robbins et al., 2019 [59]
	E3 ligase-associated regions	Immunoblot and multiplex assays	Armagan et al., 2022 [74]
**Ro60**	211-245, 255–273, 346-372	Peptide mapping, ELISA	McCauliffe et al., 1994 [72].
	169-196, 216-245	Autoantigen arrays, immunodiffusion	Schulte-Pelkum et al., 2009 [75]
	RNA-binding domain (varied)	Structural modeling and autoantibody assays	Defendenti et al., 2011 [65]
	228-237, 361-390	T cell hybridomas assays	Szymula et al., 2014 [76]

**Abbreviations:** Enzyme-Linked Immunosorbent Assay, ELISA.

Despite recent advances, the precise autoepitopes associated with SjD—particularly those presented by HLA-DRB1∗03:01—are not yet fully defined. Targeting these autoantigens, especially in combination with cell-based therapies, could enable more precise and disease-modifying treatments. Further investigation into Ro-MHC II complexes may provide critical insights into the mechanisms of autoimmunity and inform the development of targeted therapies for SjD.

## 4 Therapeutic landscape in SjD

No targeted therapy is approved for SjD. Instead, off-label medications are used to manage symptoms rather than address the underlying disease mechanisms, and none have been shown to stop progression of disease over time. Patients with *sicca* symptoms are often treated with artificial tears, medicated gels and topical fluoride, and stimulants to promote saliva production (secretagogues). For extra-glandular/systemic manifestations, management includes corticosteroids such as prednisolone and/or conventional immunomodulators/immunosuppressants such as hydroxychloroquine, methotrexate, leflunomide, or mycophenolate [77] in addition to off-label B cell depletion by rituximab for severe/recalcitrant disease and cryoglobulinaemic vasculitis. Efficacy of these treatments varies, and they do not directly address the underlying autoimmune dysregulation nor prevent disease progression, highlighting a significant gap in effective, disease-modifying therapies.

Agents targeting B cells such as, rituximab, belimumab, ianalumab, and bruton tyrosine kinase inhibitors (BTKi) have been investigated for their therapeutic potential with promising advancements (Table 5). Given the central role of autoreactive B cells in SjD, B cell depletion or targeting BAFF and/or a proliferation-inducing ligand (APRIL), which are critical for B cell survival and function [[78], [79], [80], [81]], has been a major focus. While earlier trials yielded mixed results, newer approaches are showing increasing potential. Notably, ianalumab (VAY736), a monoclonal antibody targeting the BAFF receptor to modulate B cell activity, have demonstrated encouraging results and has now progressed on a Phase 3 clinical trial. This marks a significant step towards more effective and disease-modifying treatments for SjD (Table 5).

**Table 5:** Pipeline therapy and drugs for SjD patients.

Cell Target	Biologics	Type	Target	Clinical Trial No.	Clinical Trial Phase
**B cell**	Rituximab	Monoclonal Antibody	CD20	NCT00363350 [86]	I/II
				NCT00740948 [87]	II/III
				NCT0012101829 [88]	I
				EudraCT: 2010-021430-64 [89]	III
	Belimumab		BAFF	NCT02631538 (with RTX) [90]	II
				NCT01160666 [91]	II
	Ianalumab		BAFF-R	NCT02962895 [92]	IIb
	Telitacicept	Recombinant Protein	BAFF/APRIL	NCT04078386 [93]	II
				NCT05673993 [94]	III
	Remibrutinib		BTK	NCT04035668 [95]	II
**T cell**	Frexalimab	Monoclonal Antibody	CD40L	NCT04572841 [96]	II
	Lulizumab		CD28	NCT02843659 [83]	II
	Low Dose IL-2	Recombinant Protein	Increase Treg Count	NCT02464319 [97,98]	II
	Dazodalibep		CD40L	NCT04129164 [82]	II
**SGECs**	Iscalimab	Monoclonal Antibody	CD40	NCT02291029 [84]	II
	Abatacept	Recombinant Protein	CD80	NCT02067910 (48wks) [99]	III
				NCT02915159 (24wks) [85]	III

**Abbreviation:** A Proliferating Inducing Ligand, APRIL; B cells Activating Factor, BAFF; B cells Activating Factor Receptor, BAFF-R; Bruton's Tyrosine Kinase, BTK; Regulatory T cells, Treg; Rituximab, RTX; Salivary Glands Epithelial Cells, SGECs.

In addition to B cell pathways, other strategies targeting T cell co-stimulation and their interaction with SGECs are being explored (Table 5). These therapies aim to disrupt the TCR-MHC interaction by blocking receptors such as CD40L, CD40, CD28, and CD80, pivotal for T cell activation [[82], [83], [84], [85]]. While some of these therapies have shown promise in clinical trials, results need to be confirmed in larger studies with better stratification of patients. Furthermore, additional targets to refine T cell-driven pathways remains a key objective for therapeutic development in SjD.

## 5 Potential in targeting Treg in SjD

**Fig. 2:**
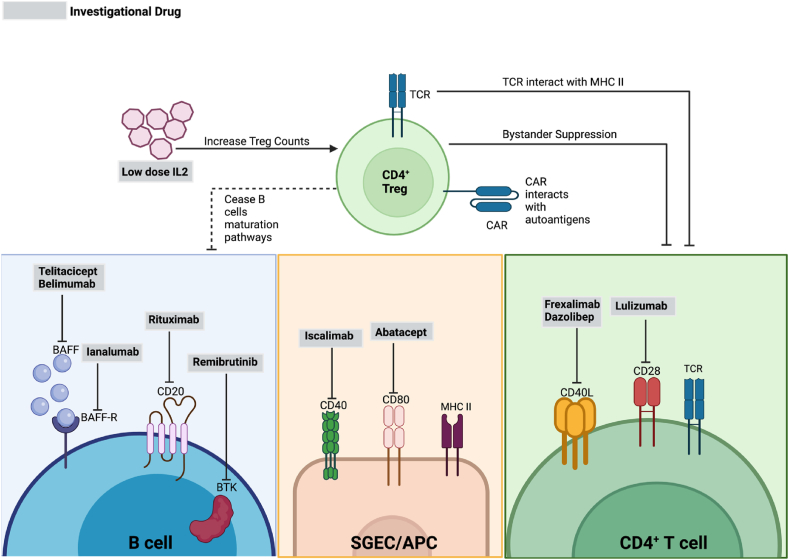
Summary of the investigational biological therapies targeting key players in SjD pathogenesis. Repurposed biologics act to inhibit the autoimmune response. Treg cell therapy posts as a potential therapeutic approach providing a more targeted therapy using CAR or engineered TCR to dampen the autoimmune response. (Image created using Biorender).

Treg play a crucial role in maintaining peripheral immune tolerance and preventing autoimmune diseases. They are characterized by high expression of CD25 and Foxp3 [[100], [101], [102]], and regulate immune responses through multiple mechanisms. Treg can induce anergy in conventional CD4^+^ T cells via CTLA-4 or PD-1 surface markers [103,104] or through TCR recognition of specific antigens presented on MHC Class II molecules [105,106]. Additionally, they provide bystander suppression to CD4^+^ T cells [104], leading to the loss of autoimmune responses towards autoantigens [102,106]. This, in turn, reduces the production of pro-inflammatory cytokines (Fig. 2), which are essential for B cell maturation and autoantibody production. Despite their critical role in maintaining self-tolerance, no clinical trials have yet explored the therapeutic potential of autologous Treg for treating SjD.

Several studies have reported impaired regulatory Treg function in patients with SjD [107]. One study demonstrated that a significantly higher proportion of Treg in SjD patients remained in a resting state compared to healthy controls, suggesting reduced cellular activation [108]. Similarly, research by Li et al. and Bianca et al. revealed a significant reduction in the number of Treg in SjD patients relative to healthy donors, with reported p-values of <0.05 and 0.00005, respectively [109,110]. Christodoulou et al. investigated FOXP3+ Treg infiltration in minor salivary glands and found a marked decrease in FOXP3+ Treg in advanced lesions compared to mild and intermediate ones [111]. These findings collectively highlight the critical role of Treg in maintaining immune tolerance to autoantigens in SjD and suggest that their dysfunction and numerical deficiency may contribute to disease pathogenesis.

Potential treatment strategies to increase the number of functional Treg are under investigation such as administration of low-dose IL-2, showing therapeutic benefit in SjD via improvement in disease activity in addition to increased numbers of Treg in a Phase II RCT (NCT02464319) (Table 5) [98,112]. The utility of low dose IL-2 therapy is yet to be confirmed, particularly with its narrow therapeutic window which may inadvertently activate pathogenic effector T cells and NK cells. Long-term effects also remain unclear, as durability of Treg expansion is yet to be confirmed.

Importantly, while enhancing Treg numbers may promote immune tolerance, this approach also raises safety concerns. Studies suggests that increasing Treg count can potentially increase the risk of malignancies due to their immunosuppressive effects, which may impair tumour surveillance [113,114]. This is particularly relevant in SjD, where patients already exhibit an increased baseline risk for the development of lymphomas, particularly non-Hodgkin's lymphomas. Consequently, the therapeutic use of Treg in SjD must be carefully calibrated. Careful consideration of the optimal dose is essential—not only to effectively restore immune tolerance and suppress autoimmune activity, but also to avoid excessive immunosuppression that may impair antitumor immunity. Balancing these factors is essential for maximizing the therapeutic benefit of Treg-based interventions while minimizing potential oncogenic risks in this patient population.

## 6 Adoptive transfer of Treg

Beyond the preferential expansion of Treg using exogenous low dose IL-2 therapy, innovative approaches such as adoptive transfer of functional Treg or other cell-based therapies offer exciting potential to restore immune tolerance. These cutting-edge strategies hold promise for achieving long-term remission and prevention of progression in autoimmune diseases, providing a more targeted and durable solution to immune dysregulation.

**Fig. 3:**
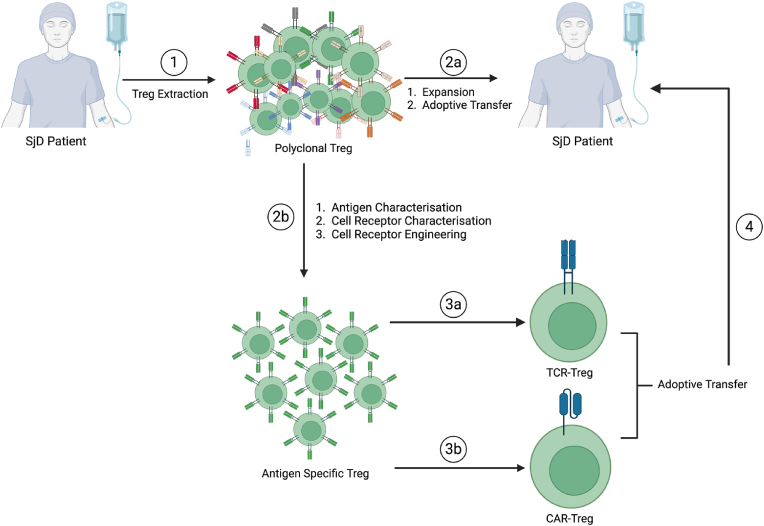
A schematic illustrating Treg therapies via autologous adoptive transfer in SjD. Polyclonal Treg are isolated from a patient's whole blood **(1)**. These polyclonal Treg can be used for adoptive transfer after reaching desired cell density (expansion) **(2a) or** undergo further characterisation for the development of antigen-specific Treg **(2b)**. Redirecting polyclonal Treg to become antigen-specific Treg allows two types of antigen-specific Treg to be engineered: T cell Receptor-Treg (TCR-Treg, **3a**) or Chimeric Antigen Receptor-Treg (CAR-Treg, **3b**). These engineered antigen-specific Treg can eventually be adoptively transferred back to the same patient (**4**). (Created using Biorender)*.*

Two types of Treg can be considered for adoptive transfer: (i) polyclonal Treg and (ii) antigen-specific Treg (Fig. 3). Antigen-specific Treg target specific antigens [115] while polyclonal Treg recognise a plethora of antigens presented by various MHC Class II molecules [116].

### 6.1 Polyclonal Treg therapy

Polyclonal Treg consist of a heterogeneous population of Treg with diverse TCRs targeting various antigens presented by MHC Class II molecules [116]. In this autologous polyclonal Treg adoptive transfer approach, Treg populations from patients are expanded *ex vivo* and subsequently adoptively transferred back [117]. Importantly, there is no genetic modification of the Treg in this process, only involving the expansion of existing Treg.

This type of approach has been studied in early Phase clinical trials of patients with type 1 diabetes (T1D) and multiple sclerosis (MS)).

In a Phase I (NCT01210664) and Phase II (NCT02691247) clinical trials for patients with T1D, no significant improvement was observed in terms of C-peptide levels, measured 1- and 2-years post-therapy [116,118]. Similarly, a Phase Ib/IIa clinical trial (EudraCT: 2014-004320-22) conducted in patients with MS showed an increase in number of polyclonal Treg, but no improvement in the Expanded Disability Status Scale (EDSS), a measure for assessing disability progression [119]. Unfortunately to date, despite promising therapeutic rationale, these trials failed to demonstrate improvements in disease outcomes, potentially due to the polyclonal nature of the Treg populations used.

### 6.2 Antigen-specific Treg therapy

One way to improve the clinical efficacy of adoptive Treg transfer is through the use of antigen-specific Treg, which can provide more targeted immune regulation. Antigen-specific Treg recognise a single epitope which is also recognised by pathogenic T cells. Two primary types of antigen-specific Treg have been developed: (i) TCR-engineered Treg (TCR-Treg) and (ii) CAR-engineered Treg (CAR-Treg). TCR-Treg express a genetically introduced TCR which recognizes a key autoepitope presented by MHC Class II molecules to pathogenic T cells [115]. CAR-Treg express a synthetic chimeric antigen receptor (CAR) which binds directly to autoantigen and is usually linked to an intracellular signalling domain which activates the Treg. In the context of SjD, intracellular autoantigens such as Ro and La become extracellularly exposed during apoptosis of salivary gland epithelial cells (SGECs), enabling their recognition by CARs designed to target autoreactive B cells.

Although no clinical trials or *in vitro* studies of antigen-specific Treg have been conducted in SjD, these approaches have been explored experimentally *in vivo* and *in vitro* for other diseases, including Haemophilia A, MS, T1D, Crohn's disease, and SLE (Table 6). These studies highlight the potential of antigen-specific Treg therapy in achieving targeted effective immunosuppression within these autoimmune models [117,127].

**Table 6:** Antigen-specific Treg therapy in autoimmune disease.

Therapy	Disease	Antigen	MHC Class II	TCR/ScFV	Delivery Methods	Refs	Mode of Testing
**TCR-Treg**	Haemophilia A	Factor VIII	HLA-DRB01∗01:01	TRAV4-1, TRBV20-1	Lentiviral Transduction	Kim et al. [120].	In vitro suppression Assay with T and B patient's cells
**CAR-Treg**		A2 epitope of Factor VIII	–	1G10 with IgG1 heavy chain	Retroviral Transduction	Yoon et al. [121].	
**TCR-Treg**	Type 1 Diabetes (T1D)	IGRP, GAD65, PPI	HLA-DRB01∗04:01	Not revealed	Lentiviral Transduction	Yang et al. [122].	*in vivo* NOD/SCID/IL2rγ^null^ mouse
**CAR-Treg**		Insulin	–	HAL9 and HAL10 libraries using mCD8 hinge	Retroviral Transduction	Tenspold et al. [123]	*in vivo* C57BL/6J non-obese diabetic mouse
**TCR-Treg**	Multiple Sclerosis (MS)	Myelin Basic Protein	HLA- DRB01∗15:01	TRAV3-1, TRBV2-1		Kim et al. [124].	*in vivo* Transgenic EAE mouse model
	Crohn's Disease	Ovalbumin	Not determined	Not determined	Culturing PBMCs with Ova peptides and using limiting dilution for clonal selection	Desreumaux et al. [125].	Phase 1b/2a clinical trial (Eudract Number: 2006-004712-44)
	Systemic Lupus Erythematosus (SLE)	Smith	HLA- DRB01∗15:01	TRAV9-2, TRBV11-2	Lentiviral Transduction	Eggenhuizen et al. [126]	*in vivo* NSG mice and suppression assay using T cells

Abbreviation: Glutamic Acid decarboxylase 65, GAD65; Islet-specific Glucose-6-Phosphatase, IGRP; Peripheral Blood Monocytes Cells, PBMCs; Preproinsulin, PPI. TCR, ScFV.

CAR T cell therapy technology has revolutionised cancer immunotherapy by enabling precise targeting and destruction of malignant cells through recognition of tumour-associated antigens [128]. Building on this concept, we postulate that activated CAR-Treg which recognise disease-specific antigens, could selectively suppress autoreactive immune responses and thus restore peripheral immune tolerance in SjD. Researchers have already identified potential variable sequences within the Ro60 autoantibody that may be leveraged for CAR-Treg therapy development. Wang et al. performed an immunoglobulin variable (IgV) peptide mapping of the native Ro60 autoantibody, revealing several variable region sequences from the serum of SjD patients [129]. Additionally, Lindop et al. identified a common light and heavy chain configuration of the Ro60 autoantibody in serum samples from seven SjD patients, with minor point mutations in the sequences [130]. These findings suggest the existence of conserved antigenic determinants that could be harnessed for antigen-specific CAR-Treg therapy.

**Fig. 4:**
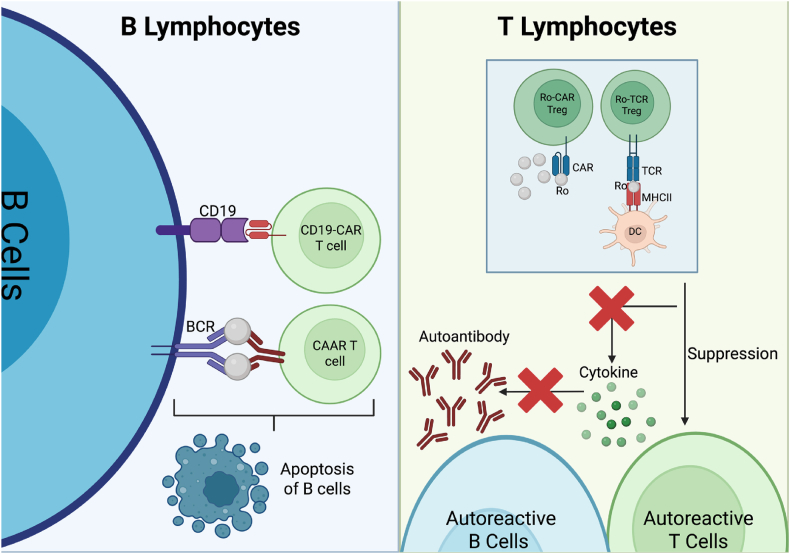
A schematic illustrating CAR and TCR therapies targeting B and T lymphocytes. CD19-directed CAR-T cells and Chimeric Autoantibody Receptor (CAAR) T cells have demonstrated therapeutic potential in autoimmune diseases by effectively depleting B cells. In contrast, CAR-Treg and TCR-Treg therapies target autoreactive T cells through immunosuppressive mechanisms, leading to the decrease of proinflammatory cytokine production and a reduction in autoantibody generation. Notably, CAR-based approaches generally require a high antigen density on target cells to elicit a robust therapeutic response. (Figure created using Biorender).

In parallel, the application of conventional CAR T cell therapy has already shown promise in SjD. Anti-CD19 CAR T cell therapy demonstrated notable therapeutic efficacy in a patient with SjD. Following treatment, the patient's anti-Ro52 autoantibody levels declined to undetectable levels, circulating pro-inflammatory cytokine concentrations normalized, and clinical symptoms associated with glandular dryness showed marked improvement [131]. These results highlight the therapeutic potential of CAR T cell therapy in targeting CD19^+^ B cells in SjD, similar to B cells depletion therapy (Fig. 4). Moreover, this strategy may provide a foundation for developing CAR-engineered Treg therapies for SjD, offering a more precise and immune-protective alternative to current treatments that indiscriminately deplete all B cells. Instead, this approach aims to preserve overall B cell function by specifically suppressing autoreactive T cells (Fig. 4).

Although CAR-based approaches have demonstrated significant therapeutic potential, their effectiveness often depends on high antigen density to achieve a meaningful therapeutic response [132]. In contrast, TCR require only recognition of peptide–MHC complexes to initiate an immunosuppressive response, potentially allowing for more efficient targeting of autoreactive cells even at lower antigen densities (Fig. 4).

A key challenge in implementing antigen-specific Treg therapies lies in addressing epitope spreading—a phenomenon whereby multiple autoantigens drive the activation of diverse autoreactive T cell clones [133]. Thus, selecting epitopes with well-characterized autoantigenicity and clear pathogenic relevance is critical to ensure targeted and effective immunoregulation. Importantly, a study in autoimmune arthritis have shown that Treg depletion exacerbates epitope spreading in both T and B cell compartments [134], highlighting the central role of Treg in maintaining immune homeostasis. Collectively, these findings support the therapeutic value of Treg-based approaches and underscore their potential utility in treating SjD.

## 7 Conclusion

SjD presents a significant challenge in treatment due to its heterogenous and systemic nature. It is a disease that has impact not just on exocrine gland function. Current therapeutic strategies rely on a variety of treatment strategies that manage the symptoms, as well as the long-term off-label use of conventional and biological immunosuppressants and corticosteroids, that reduce severity of systemic inflammation. However, these treatments have variable effectiveness and do not directly address the underlying pathogenic mechanisms of SjD.

Encouragingly, an active pipeline of RCTs exploring novel therapeutic approaches have shown promising results, with progression to Phase 3 studies. A particularly exciting potential avenue is the use of Treg therapy, which offers a more targeted and potentially transformative approach by precisely regulating autoimmune responses and restoring immune tolerance in SjD. Advances in bioengineering and cell therapy techniques are paving the way for innovative Treg based treatments, bringing new hope for patients seeking more effective, safe and durable solutions. targeting of autoantigen in SjD and a more targeted and potentially potentially transformative approach for treating SjD. Different ways to utilising Treg, including some state-of-the-art bioengineering advancement, is currently being studied in order to develop new therapies for patients with SjD.

## CRediT authorship contribution statement

**Zhi Feng Sherman Lim:** Writing – review & editing, Writing – original draft, Validation, Formal analysis, Conceptualization. **Alberta Y. Hoi:** Writing – review & editing, Writing – original draft, Conceptualization. **Fabien B. Vincent:** Writing – review & editing, Writing – original draft, Supervision, Conceptualization. **Joshua D. Ooi:** Writing – review & editing, Supervision. **Eric F. Morand:** Writing – review & editing. **Maureen Rischmueller:** Writing – review & editing, Writing – original draft, Supervision. **Yi Tian Ting:** Writing – review & editing, Writing – original draft, Validation, Supervision, Formal analysis, Conceptualization.

## Ethics approval and consent to participate

N/A.

## Availability of data

N/A.

## Financial support information

This work was supported by the 10.13039/501100000925NHMRC, Australia, and the Long Foundation. FBV is supported by funding from 10.13039/501100000925National Health and Medical Research Council (Emerging Leadership 1, grant 1196112) and has received support from the Rebecca L Cooper 10.13039/501100009187Medical Research Foundation and 10.13039/501100000940Arthritis Australia. FBV has received support from 10.13039/100015756Janssen-Cilag, 10.13039/100004325AstraZeneca and CSL for projects, and from 10.13039/100004319Pfizer, 10.13039/100012051Lupus Research Alliance, and SomaLogic for conference or meeting sponsorship.

## Declaration of competing interest

The authors declare the following financial interests/personal relationships which may be considered as potential competing interests: Associate Professor Joshua Ooi, Dr Fabien Vincent reports financial support was provided by 10.13039/501100000925National Health and Medical Research Council. If there are other authors, they declare that they have no known competing financial interests or personal relationships that could have appeared to influence the work reported in this paper.

## Data Availability

No data was used for the research described in the article.
